# The role of seminal reactive oxygen species assessment in the setting of infertility and early pregnancy loss

**DOI:** 10.1007/s00345-023-04472-2

**Published:** 2023-07-15

**Authors:** Rhianna Davies, Suks Minhas, Channa N. Jayasena

**Affiliations:** 1 Department of Metabolism, Digestion and Reproduction, Imperial College London, London, UK; 2 Department of Urology, Charing Cross Hospital, Imperial College NHS Trust, London, UK

**Keywords:** Infertility, Recurrent pregnancy loss, Reactive oxygen species, ROS, Infertility, Semen

## Abstract

The male contribution to a couple suffering with adverse early pregnancy outcomes is being increasingly investigated. Seminal oxidative stress is considered to cause sperm DNA damage, thus affecting the functional capacity of the sperm. Multiple lines of evidence support an association between elevated seminal reactive oxygen species (ROS) and infertility. In the setting of assisted reproduction various factors in the in vitro environment, differing from the in vivo environment, may exacerbate oxidative stress. Furthermore, seminal ROS levels have been found to be higher in the male partners of couple’s affected by both spontaneous and recurrent pregnancy loss. There are several methods by which to assess ROS levels however they are costly, inconsistent and their incorporation into clinical practice is unclear. The value of ROS assessment lies in the ability to plan targeted therapies to improve pregnancy and live birth rates. As such, further robust study is required before firm conclusions can be made to inform clinical practice. We aim to review the available evidence regarding the role of seminal ROS in infertility and pregnancy loss.

## Introduction

Reproductive complications can have devastating effects on a couple [1]. Historically the burden of investigation and intervention has laid with the female. Increasingly the male contribution to a couple suffering reproductive difficulties is being explored. Semen analysis remains the cornerstone of investigation for male infertility; this assesses concentration, motility and morphology of the sperm but does not establish their functional capacity [2]. Recent advances have led to a greater appreciation of the importance of the integrity of the DNA carried by sperm. A proposed mechanism for sperm DNA damage is by the action of reactive oxygen species (ROS) within the seminal fluid [3–5]. There is currently no clinical guidance regarding the use of ROS measurement in the routine assessment of the infertile male [6]. This review critically appraises the value of seminal ROS measurement in the evaluation of a couples suffering from infertility and early pregnancy loss.

## Physiological seminal ROS

Containing at least one oxygen atom, ROS are extremely unstable and capable of extracting an electron from surrounding molecules to achieve an electronically stable state. The surrounding molecule that has lost an electron is now at risk of being an unstable ROS itself, instigating a chain of ROS generation [7, 8]. Spermatozoa are abundant in mitochondria due to their great demand for motile energy [9]. Nicotinamide-adenine-dinucleotide-phosphate (NADPH)-dependant oxidoreductase processes within both the mitochondria and the plasma membrane of spermatozoa generate superoxide [10–12]. ROS are physiologically generated within the male reproductive tract. Superoxide represents the largest contributor to ROS within the human spermatozoa [7, 8]. Furthermore, seminal leucocytes are also capable of generating ROS [4]. Seminal ROS are physiologically required to aid sperm motility, capacitation, the acrosome reaction and fertilisation of oocytes [4] (Table 1).

**Table 1:** Physiological roles of seminal ROS [96–98]

Sperm motility	Downstream activation of protein tyrosine kinase which phosphorylates tyrosine residues in the sperm cytoskeleton Inhibition of phosphotyrosine phosphate from dephosphorylating tyrosine residues
Capacitation	Downstream activation of protein tyrosine kinase which phosphorylates tyrosine residues in the sperm cytoskeleton Inhibition of phosphotyrosine phosphate from dephosphorylating tyrosine residues
Acrosome reaction	Induction of phosphorylation of plasma membrane proteins to facilitate the release of acrosomal enzymes to penetrate the zona pellucida
Fertilisation	Inhibition of deactivation of phospholipase A2. Phospholipase A2 cleaves fatty acids from the plasma membrane which increases fluidity allowing fusion between the sperm and oocyte

To ensure a balance is maintained between physiological oxidative actions, reducing agents are also produced to minimise cellular injury [13]. These reducing agents are termed antioxidants and can be both endogenously produced and exogenously consumed via diet. The seminal vesicles, bulbourethral glands and prostate secrete antioxidants into seminal fluid [14]. In vitro experiments removing the seminal plasma surrounding the sperm found reduced motility and increased oxidative stress within 2 h [15]. Exogenous examples of anti-oxidants include ascorbic acid (vitamin C) that neutralises ROS and zinc which may protect against lipid oxidation [16].

## Pathological seminal ROS

Abnormal spermatozoa and activated leucocytes are considered the major contributors to seminal ROS [4]. Leucocytes may be activated by infection or inflammation, and evidence suggests an activated leucocyte produces 100 time more ROS than its inactivated counterpart [17, 18]. Excess ROS generation, occurring through both exogenous and endogenous pathways, may be caused by physiological stress, environmental pollutants, thermal stress, lifestyle factors, aging, infection, varicocele and medical comorbidities [19–26]. In vitro studies of human sperm established that ROS can induce decreased motility by virtue of peroxidation of lipids in the sperm membrane which impairs the flexibility of sperm required for movement, and also by damaging the mitochondria and thus energy-producing capacity of the sperm [27, 29]. Human studies have also shown an association between oligospermia and higher levels of ROS due to disruption of mitochondrial membranes and thus activation of caspases [29] (Table 2). Caspases induce apoptosis [17]. Apoptosis itself induces the release of cytochrome c which promotes the generation of further ROS [30]. This represents a cycle of ROS generation (see Table 3).

**Table 2:** Mechanisms of ROS-induced sperm damage [99]

DNA	Direct injury to purines/pyrimidines Chromosomal rearrangement Single-strand breaks Double-strand breaks Cross-linking
Mitochondria	Damage to mitochondrial DNA Decreased ATP production Caspase release from damaged mitochondrial membrane
Cell membrane	Lipid peroxidation of polyunsaturated fatty acids

**Table 3:** Examples of commonly used techniques to assess oxidative stress [6, 49, 75]

	Name	Technique	Advantages	Disadvantages
Direct:	Chemiluminescence assay	Detection of oxidation or reduction through the by-product generation of light to measure ROS	Robust High sensitivity and specificity It can measure both intra- and extracellular ROS	Time-consuming Expensive (equipment) Variables such as the volume of semen may affect measurement
Indirect:	Lipid peroxidation level	Levels of malondialdehyde (MDA) by-products of lipid peroxidation are measured by thiobarbituric acid assays by colorimetry or fluoroscopy	Can be used for frozen samples	Non-specific
	The Male Infertility Oxidative System (MiOxsys)	Measurement of the transfer of electrons from reductant to oxidant in millivolts	Fast Can be used for both semen and seminal plasma Can be used for frozen samples Cost-effective	Affected by the viscosity of the sample
	Total antioxidant capacity (TAC)	An enhanced chemiluminescent assay which measures the suppression of chemiluminescence in seminal plasma and the time to recovery	Fast Measures the total antioxidants in seminal plasma	Does not measure enzymatic antioxidants Expensive (microplate readers)

### Infertility

Male factor infertility accounts either exclusively or jointly for 50% of all couples struggling to conceive, and yet the exact causative factor is identified in only 30–50% of these men [29, 31]. Such idiopathic male infertility, i.e. abnormal seminal parameters without identifiable cause and the absence of female factor infertility, precludes specific therapy [32]. Multiple lines of evidence from human studies support the hypothesis that seminal oxidative stress plays a role in male factor infertility [33–39]. The detrimental effects that ROS have on sperm morphology and motility have been widely noted [29]. Indeed, depending upon the ROS assay used studies show that 30–88% of unselected infertile men have elevated seminal ROS [7, 37–48]. As such, the term Male Oxidative Stress Infertility (MOSI) has been introduced to describe men with idiopathic infertility and elevated seminal ROS [49]. The role of seminal ROS testing in unexplained male factor infertility, i.e. normal semen parameters, is less clearly defined. However, evidence suggests that in the presence of normal semen parameters seminal ROS levels may still be elevated such to have deleterious effects on fertilisation capacity [50]. Pasqualotto et al. enrolled 42 normospermic infertile men and 19 healthy controls into a study investigating ROS levels in the setting of unexplained infertility [50]. They found the infertile men, despite normal semen parameters, had significantly higher ROS levels compared with controls (*p* < 0.004). They also had lower TAC scores than healthy controls [50]. Indeed, the levels of ROS needed to impair, for example, motility may be higher than the levels of ROS needed to impair sperm-oocyte fusion [51]. Furthermore, the aetiology of the deleterious effects of a varicocele, considered to potentially account for a large portion of both primary and secondary infertility, is thought to be due to the generation of ROS [47].

### Assisted reproductive techniques (ART) outcomes

It has been identified that oxidative stress contributes to suboptimal outcomes in assisted reproduction [52]. A meta-analysis by Agarwal et al. included 3 studies (*n* = 122) investigating the relationship between ROS levels, measured via chemiluminescent assay and fertilisation rates during human in vitro fertilisation (IVF) [51]. This encompassed couples with various causes of infertility including male factor, female factor and unexplained infertility. They observed a negative correlation between seminal ROS and fertilization rates [51]. A study by Hammadeh et al. recruited 48 men from the setting of infertility services [53]. Twenty-six of the enrolled men were from couples undergoing IVF and 22 undergoing intracytoplasmic sperm injection (ICSI). ROS was measured using the BioMedica Oxy Stat assay which measures the total amount of peroxides in EDTA plasma (reference value < 400 μmoll/l). In both groups a negative correlation was found between ROS levels and sperm morphology and vitality, and importantly, also fertilisation rates [53]. The source of ROS can be both from the gametes and the exogenous environment including the culture medium, light exposure, pH differences, temperature differences [52, 54]. The processes of ART including centrifugation, cryopreservation and thawing can induce the generation of ROS [54]. Furthermore, within the in vitro setting the absence of the physiological reducing mechanisms to buffer for ROS is absent [54]. As within the in vivo environment, immature spermatozoa and seminal leukocytes generate ROS [4, 10–12]. The in vitro manipulation of sperm exposes them to further ROS for example the longer incubation periods required during IVF results in exposure to ROS for longer [55, 56].

There is a direct correlation between centrifugation time and ROS generation, likely secondary to thermal exposure [57, 58]. Shekariz et al. investigated the association between centrifugation and ROS generation compared the semen samples of 24 infertile men and 14 normal donors [57]. They measured ROS using a chemiluminescent assay and classed ‘high’ ROS as at least 10 × 10 (4) counted photons/min (cpm). They measured ROS before centrifugation, after 2 min of centrifugation and after 10 min of centrifugation. Eight participants (7 infertile men, 1 control) had elevated ROS in their pre-centrifugation measurement; all 8 of these men demonstrated a significant increase in ROS levels following centrifugation at both 2 min and 10 min. The elevated in ROS was significantly more following 10 min of centrifugation compared with 2 min. Furthermore, 6 specimens who had previously had low seminal ROS had high seminal ROS following 10 min of centrifugation [57]. In vitro studies of human sperm suggest that cryopreservation risks damage to the sperm cell membrane resulting in the production of ROS [59]. The medium within which the embryos are cultured in directly affects outcome [55]. Bedaiwy et al. undertook a prospective study of men undergoing ART; they enrolled 104 men from a couple having IVF and 91 from a couple having ICSI [56]. They measured day 1 ROS levels via a chemiluminescent assay in both the central well and an outer well of the embryo culture dish. The central wall acted as the sample and the outer well as a control. They found that high day 1 ROS levels in the culture were associated with lower clinical pregnancy rates in both the IVF and ICSI groups. Interestingly they found that whilst elevated ROS levels in the culture medium negatively correlated with blastocyst development and fertilisation rates in the ICSI group, the same was not observed in the IVF group [56]. Metallic ions, in particular, present in culture medium induce free radicals production by the embryo [60]. Similarly, visible light can induce embryonic ROS production [54]. A study by Takenaka et al. found that the live birth rate was greater in the zygotes shielded from light than those exposed to white fluorescent lights for as little as 15 min [61]. In physiology, early embryo development occurs in an environment of low oxygen tension [56]. It is known that an aerobic environment increases the generation of ROS [54]. A study by Bavister et al. found significantly higher DNA damage in embryos cultured at atmospheric oxygen levels compared with those cultures at lower oxygen tensions [62].

ICSI increases the risk of oxidative stress by 2 discrete mechanisms [54, 63]. Firstly, the lack of natural selection of quality sperm may result in sperm with a greater rate of ROS-induced DNA damage being injected into the oocyte [55]. Secondly, when the sperm is injected into the oocyte some of the surrounding ROS-containing medium will be injected alongside it [63]. Elevated ROS levels in culture medium during ICSI have been shown to be associated with higher embryonic DNA fragmentation, a lower cleavage rate and a lower fertilisation rate [63]. There is a longstanding understanding that there is an association between duration of sexual abstinence and parameters on routine seminal analysis [64]. A randomized control trial by Degirmenci et al. (2020) established that longer periods of sexual abstinence cause greater ROS and lower pregnancy rates in intra-uterine insemination (IUI) cycles [65].

Thus, evidence suggests that oxidative stress plays a role in adverse ART outcomes in some cases [51, 52]. Various ART-associated factors may induce ROS generation such as the culture medium, light and temperature, centrifugation and cryopreservation. Furthermore, the in vitro environment is lacking in the antioxidant effects present in vivo [52] [54]. The exact differences in outcome between the various types of ART with regard to oxidative stress are yet to be clearly elucidated [56].

### Miscarriage

Several lines of evidence suggest a role of ROS-induced sperm DNA fragmentation in the aetiology of miscarriage. A study by Kamkar et al. found a significantly higher amount of seminal ROS and a lower total antioxidant capacity (TAC) in the male partners of women who had experienced a spontaneous miscarriage compared to the male partners of women who had not; sperm DNA fragmentation was also increased in the affected group [2]. To reinforce this, further study has investigated a potential association between elevated seminal ROS and recurrent miscarriage (recurrent pregnancy loss; RPL), defined as 2 or more miscarriages under 24 weeks of gestation. A study by Jayasena et al. demonstrated a fourfold increase in seminal loss levels in the male partners of women affected by RPL than the control arm [66]. A study by Venkatesh et al. studied established evidence of increased levels of ROS and DNA sperm damage in the male partner of a women affected by RPL compared with controls [67]. Imam et al. compared ROS levels via a chemiluminescent assay in 20 men with a history of PRL with 20 healthy controls [68]. They found significantly higher levels of ROS and sperm DNA damage in the RPL compared with the control group. Furthermore, TAC was significantly lower in the RPL group than in the control group. Gil-Villa et al. analysed semen from 23 men whose partners had experienced RPL and 11 control men with recent established fertility [69]. They found that the RPL group had higher levels of lipid peroxidation and a lower TAC than controls [69].

In summary, evidence suggests that oxidative stress may also be implicated in miscarriage [51, 52]. The association between elevated seminal ROS and sperm DNA damage, and miscarriage reinforces the hypothesis that ROS-induced DNA damage may increase the risk of both spontaneous and recurrent pregnancy loss.

## Assessment of oxidative stress

A variety of tests may be used to ascertain the oxidation–reduction balance; these can be subdivided into direct and indirect tests [39, 49]. Direct tests measure ROS production and oxidation, whilst indirect tests measure the subsequent markers of ROS-induced injury [39] (Table 1). Direct tests include chemiluminescence assays whereby charged and uncharged probes undergo either reduction or oxidation, and light is produced during the reaction. This ROS assay is the most commonly utilised direct method of assessment [6]. Using a luminol-based chemiluminescence assay in 258 infertile men and 92 controls Agarwal et al. sought to establish a cut-off value to differentiate between fertile and infertile. They observed that a cut-off of 102.2 RLU/s/10^6^ was 76.4% sensitive, with a positive predictive value or 82.1%, with respect to fertility vs infertility [70]. Indirect tests include TAC which establishes the ability of antioxidants present in a sample to scavenge free radicals [70]. Using a cut-off value of 1947 µm, Roychoudhury et al. found TAC assessment gave a specificity of 63% and sensitivity of 59.5% to distinguish between fertile and infertile [71]. Another indirect measurement technique is to establish the degree of lipid peroxidation as a surrogate for ROS-induced damage to the sperm lipid membrane. The by-product of lipid peroxidation, malondialdehyde (MDA), is measured by colorimetry [6]. Table 1 summarises the commonly used methods, and their relative advantages and disadvantages (Table 1). These tests may be difficult to incorporate into routine practice as many are expensive, time-consuming and require extensive training [49]. Additionally, the results of samples assessed by the various tests available do not correlate well with each other [72].

The more recent oxidation–reduction potential (OPR) testing via the Male Infertility Oxidative System (MiOxsys) shows great promise [49]. It is considered a fast, inexpensive and reproducible method that is more accurate than chemiluminescent assessment of ROS [73]. The process takes 4 min and liquefaction is the only sperm preparation required [6]. This method establishes the relative proportion of oxidant and antioxidant activity within a sample; a high OPR score indicates an oxidative stress predominant environment [74]. Mounting evidence suggests correlation may exist between the OPR level and traditional semen parameters; elevated OPR is associated with a greater likelihood of abnormal total count, motility and morphology [74]. An assessment of 366 men via both traditional semen analysis and ORP found significantly lower OPR values in men with normal semen parameters (*p* < 0.0001) [75]. Furthermore, OPR can be used for cryopreserved samples to predict ART success [75]. However, OPR is affected by semen viscosity and it is an extracellular marker, whilst sperm DNA damage is intracellular [36, 76]. As oxidative stress generates sperm DNA damage overtime rather than immediately, OPR is not an independent measure of ROS-induced functional impairment of sperm [76].

## Clinical application

There are no clinical guidelines to direct the use of these tests in the assessment of a couple suffering from infertility or early pregnancy loss. There are certain markers in traditional semen analysis which may suggest high ROS levels and trigger testing, for example the presence of asthenozoospermia (reduced motility) or leucocytospermia [77].

### Oral antioxidants

Many authors support the selective or empirical treatment of subfertile men with oral antioxidants on the premise that their infertility is driven by elevated ROS levels not counteracted by the reducing capacity of the antioxidants in seminal fluid. Such antioxidants include vitamins A, B, C, E, Coenzyme Q10, l-carnitine and alpha-lipoic acid. A blinded, placebo-controlled trial by Haghighian et al. randomised men with idiopathic asthenozoospermia to either alpha-lipoic acid supplementation or placebo for 12 weeks (*n* = 44) [78]. They found significant improved sperm concentration, count and motility as well as increased seminal TAC levels in the treatment group, which was not replicated in the control group [78]. A further blinded randomised and placebo-controlled trial by Nadjarzadeh et al. found increased seminal TAC, decreased ROS levels, but no change in traditional semen parameters in men with idiopathic oligoasthenoteratozoospermia (sperm that are abnormal in count, motility or shape) treated with Coenzyme Q10 [79]. A 2018 meta-analysis suggested antioxidant use improves sperm quality and ART outcomes [80]. A 2019 Cochrane review also found reproductive benefit with the use of antioxidant therapy [81]. A 2021 prospective cohort study which enrolled 44 men with abnormal traditional sperm parameters found improvement in sperm count and motility following antioxidant therapy in the group with elevated ROS but not in the group without elevated ROS [82]. They used an in-house luminol-based colorimetric assay and classed > 10 RLU/s/10^6^ as elevated [82]. Agarwal et al. (2021) published clinical guidance supporting the use of antioxidants in men with unexplained infertility, abnormal semen analysis or a varicocele [83]. However, the MOXI study (Males, Antioxidants and Infertility), a multicentred, double-blind randomised control trial, failed to demonstrate any improvement in semen parameters, DNA sperm damage or pregnancy rates with the use of oral antioxidants [84]. It is worthy of note that the clinical efficacy of antioxidant therapy for male infertility is yet to be clearly elucidated, and indeed high levels of antioxidants may induce reductive stress [85]. For example, increased seminal plasma levels of selenium have been found to be associated with decreased sperm motility and increased miscarriage rates [86]. A delicate balance in the redox system is required for normal embryogenesis. Menezo et al. found increased rates of sperm chromatin decondensation following 90 days of antioxidant treatment; this has potential ramifications on paternal gene activity in the pre-implantation embryo [87]. Well-designed randomised control trials, adequately powered to assess for an association between antioxidant supplementation and liver birth rate, are warranted before definitive conclusions can be drawn.

### Varicocele repair

Some studies support the value of varicocele repair in the improvement of male infertility, though these results are not universally replicated and thus guidelines vary [88–90]. The European Association of Urology (EAU) advocate varicocele repair in men who have all 3 of the following: clinical varicocele, oligozoospermia and subfertility [31]. Guidelines published by both the American Urological Association (AUA) and the American Society for Reproductive Medicine (ASRM) recommend that all of the following 4 criteria are met: the varicocele is palpable on examination, the presence of subfertility, abnormal semen parameters and the absence of female factor infertility [91, 92]. The 2013 National Institute for Health and Clinical Excellence (NICE) guidance recommended that varicocele repair only in the setting of obstructive azoospermia and prior to sperm retrieval for ART [93]. Chen et al. assessed sperm DNA damage and antioxidant capacity in men with subfertility and a varicocele before and after varicocele repair [48]. They found that following varicocele repair there was significantly lower sperm DNA damage and increased antioxidant capacity. More than 70% of patients had improved sperm motility and morphology post-operatively [48]. Dada et al. studied men with infertility, varicocele and elevated seminal ROS [47]. They found ROS levels rapidly declined (within 1 month), whilst sperm DNA damage declined at a slower but significant rate post-operatively [47]. A 2007 meta-analysis by Marmar et al. found that post-operative spontaneous pregnancy rates were increased in men with both varicocele and at least one abnormal parameter on routine analysis [88]. They concluded abnormal semen analysis in the presence of a varicocele was an indication for surgery in an infertile male [88]. A more recent meta-analysis by Esteves et al. (2015) found improved pregnancy rates with ICSI in men with a varicocele who underwent repair compared to those with an unrepaired varicocele [90]. In contrast, Baazeem et al. failed to observe an improvement in spontaneous pregnancy rates following varicocele repair [89]. Varicocele repair to improve male fertility is controversial.

### Amendments to ART regimes

As described above evidence suggests the environment including temperature, oxygen tension and illumination affect ROS production [54, 56]. During ART embryos are cultured at atmospheric oxygen tensions (20%). This is in contrast to physiology whereby early embryo development occurs in the relatively hypoxic environment of the fallopian tubes and uterus (2–8% oxygen concentration) [94]. As such efforts are made during the ART process to mimic this low oxygen tension state by reducing the exposure of gametes and embryos to air [56]. Whilst the evidence for the effect of visible light on in vitro embryos has only been established in animals, extrapolating to humans would suggest ART outcomes could be improved by shielding developing embryos from light [54]. The identification that metallic ions induce ROS generation in culture medium has led to the addition of metal chelators in culture medium [60, 95]. Indeed, the additional of transferrin, a chelator of iron, has been found to reduce ROS [95]. The possibility of antioxidant use in ART in certain clinical scenarios is uncertain [54].

## Conclusion

An enlarging body of evidence supports the contribution of elevated seminal ROS to the aetiology of adverse early pregnancy outcomes [33–39]. There are a variety of tests to either directly measure ROS levels, measure the antioxidant capacity of a sample or indirectly measure ROS by the assessment of markers of ROS-induced injury [39, 49]. However, there is conflicting evidence regarding the clinical applicability of these tests [49]. At the present time the *World Health Organisation Laboratory Manual for the Examination and Processing of Human Semen* states that due to variation between machines and methodologies and the low quality of evidence for prognosis, there are no evidence-based reference limits for seminal ROS [100]. Furthermore, the value of assessment of seminal ROS lies in the potential ability for this to inform the development of targeted treatment strategies. Whilst the literature describes various methods to overcome the deleterious effects of oxidative stress on early pregnancy outcome, there remains a lack of clear evidence-based guidance to inform clinical practice. Further robust study to establish the value of ROS assessment and subsequent targeted therapy for male infertility would be welcomed to inform clinical guidance and practice.

## Data Availability

Not applicable.
